# Comparison of immune response generated against Japanese encephalitis virus envelope protein expressed by DNA vaccines under macrophage associated versus ubiquitous expression promoters

**DOI:** 10.1186/1743-422X-8-382

**Published:** 2011-08-02

**Authors:** Mohammad Feraz Ahsan, Milind M Gore

**Affiliations:** 1 National Institute of Virology, Pashan Campus, 130/1, Sus Road, Pashan, Pune, India

## Abstract

**Background:**

Japanese encephalitis virus (JEV) is the leading cause of viral encephalitis, with ~50,000 cases reported annually worldwide. Vaccination is the only measure for prevention. Recombinant vaccines are an efficient and safe alternative for formalin inactivated or live attenuated vaccines. Nowadays, incorporation of molecular adjuvants has been the main strategy for melioration of vaccines. Our attempt of immunomodulation is based on targeting antigen presenting cells (APC) "majorly macrophages" by using macrosialin promoter. We have compared the immune response of the constructed plasmids expressing JEV envelope (E) protein under the control of aforesaid promoter and cytomegalovirus (CMV) immediate early promoter in mouse model. Protection of immunized mice from lethal challenge with JEV was also studied.

**Results:**

The E protein was successfully expressed in the macrophage cell line and was detected using immunofluorescence assay (IFA) and Western blotting. APC expressing promoter showed comparable expression to CMV promoter. Immunization of mice with either of the plasmids exhibited induction of variable JEV neutralizing antibody titres and provided protection from challenge with a lethal dose of JEV. Immune splenocytes showed proliferative response after stimulation with the JEV antigen (Ag), however, it was higher for CMV promoter. The magnitude of immunity provided by APC dominant promoter was non-significantly lower in comparison to CMV promoter. More importantly, immune response directed by APC promoter was skewed towards Th1 type in comparison to CMV promoter, this was evaluated by cytokine secretion profile of immune splenocytes stimulated with JEV Ag.

**Conclusions:**

Thus, our APC-expressing DNA vaccination approach induces comparable immunity in comparison to ubiquitous promoter construct. The predominant Th1 type immune responses provide opportunities to further test its potency suitable for response in antiviral or anticancer vaccines.

## Background

JEV belongs to the family *Flaviviridae*. It is transmitted to humans by mosquitoes leading to the infection of central nervous system and encephalitis. JEV has covered a vast geographic area of Asia and parts of Oceania [1]. Nearly half of the human population falls in countries where JEV occurs, globally 50,000 cases are reported with 15,000 mortality rate per year [2-5].

Vaccination is the only way in controlling JEV outbreaks. Several such vaccines have been used with considerable success. The only WHO recommended vaccine used worldwide was BIKEN which was a formalin inactivated vaccine from infected mouse brain. Live-attenuated JE vaccine (SA 14-14-2) prepared in infected primary hamster kidney cells is used in China for many years and is in use by other countries like India and Nepal in recent times. Recently, Vero cell derived inactivated JE vaccine has also been licensed. Chimeric Yellow fever-JE vaccine is undergoing phase III trial [6]. Each of these vaccines have their own drawbacks [7,8], and as such there is a need for the development of safer and cost effective vaccine with higher potency which can elicit both the arms of immune response, such as DNA vaccines [9].

JEV is a single stranded, positive sense RNA virus. The genomic RNA is ~11 kb with single open reading frame (ORF) that encodes structural protein (capsid (C), premembrane (prM) and E) followed by seven non-structural protein (NS1 to NS5) [10,11]. E protein plays a major role in the infection, such as receptor binding and membrane fusion [12]. E protein induces virus neutralizing antibodies and these have been shown to neutralize virus activity through passive administration in mice model also [13]. For proper folding of E protein, co-synthesis of prM protein is required [14]. Subvirus particle with only prM and E protein has also generated protection against lethal JEV infection [15]. DNA vaccine encoding E protein is considered to be highly effective in providing protective immunity when compared with other proteins of JEV [16].

With the growing knowledge of molecular information on JEV, recombinant vaccines using various approaches [17] with different gene products [18-20] have been tried. Such vaccines have shown considerable success albeit with some shortcomings; either in terms of evoking suboptimal response or not maintaining the balance between Th1 and Th2 response [21]. Therefore the present attention has shifted towards the improvement of DNA vaccine modulated through several immunological adjuvants, such as the use of liposomes [22], inclusion of CpG motif [23], co-expressing cytokines and costimulatory molecules along with the target gene [24], exploring different routes of administration of vaccine [25-27], targeting the vaccine to specific cells [28] or endosomal/lysosomal compartment [29].

One such optimization is to target the antigen expression in professional APC by using promoters active only in APC [30]. Dendritic cell (DC) as an APC have preference over macrophage and B cells as a potent cell in priming and stimulating naïve T cells. Langerhans cells have been targeted using Dectin-2 promoter [31]. For the treatment of HIV-1, APC have been targeted [32]. Lentiviral vectors were used to deliver the gene into APC [33].

Immune response to any antigen is a highly intricate and balanced mechanism. To prevent unwanted immune responses like autoimmunity, hypersensitivity and to induce long term antigen specific immunity, specific cells with appropriate cytokine and cell surface antigen milieu have been devised by immune system. Presentation of antigen through APC would thus be the most desirable approach while developing newer vaccine. Although, DC specific promoter has shown promising results, meagrely targeting DC was reported to be insufficient to optimally induce T cell immunity [34,35]. Therefore the role of other professional APC (Macrophage and B-cells) needs to be considered. Studies suggest macrophages are potent enough to stimulate naïve CD8+ T cells to proliferate and mature [36] and could be as good as DC in cross presentation of antigen [37]. Thus there is a need to explore promoters which could be active also in other cells of APC and just not a single population.

Our earlier work for initial screening of promoters was carried out in macrophage and non-macrophage cell lines at the level of mRNA and protein using GFP as a reporter system [38]. Briefly, three promoters were selected based on their known expression profiles. Macrosialin, is a glycoprotein expressed specifically in murine monocytes and macrophages and to a lesser extent by DC [39-41]. Activity of this promoter along with two other promoters; Emr-1 [42-44] and Beta-5 Integrin [45,46], was compared with immediate early promoter of CMV. Macrosialin was chosen for further studies as it showed the highest expression amongst the APC expressing promoters albeit to a lesser extent in comparison to CMV promoter.

To study the effect of APC dominant expression as against ubiquitous expression on protective immune response, JEV system has been used. We report here studies carried out by immunizing mice with plasmids expressing JEV E protein under macrosialin promoter and comparing it with CMV promoter in terms of protective immunity and immune balance.

## Methods

### Virus and Antigen

JEV strain 733913 was used in all experiments [47]. Virus pools were prepared for *ex vivo *experiments in porcine stable kidney (PS) cells. For *in vivo *experiment two days old infant mouse brain derived virus pool suspended in 0.75% bovine serum albumin (BSA) in PBS was used. Virus stocks were stored at -70 in aliquots and titrated using plaque assay and in two months old mice group respectively. Mouse brain antigen (MbrAg) for mice inoculation was prepared in borate saline containing 8.5% sucrose, homogenized and inactivated with β-propiolactone. For *ex vivo *studies, cell culture derived virus antigen as cell slurry was used [48].

### Cell Culture

PS cells (National Center for Cell Sciences (NCCS), Pune, India) were maintained in minimal essential medium (MEM) (Sigma) supplemented with 2 mmol/l L-glutamine and 10% fetal bovine serum (FBS) (Gibco, USA). Whereas RAW264.7 cells (NCCS, Pune, India) were maintained in high glucose DMEM with 10% FBS (Gibco, USA) supplemented with penicillin (100 IU/ml) and streptomycin (100 μg/ml) at 37°C with 5% CO2 in humidified environment. For all transfections studies, cells were maintained without antibiotics.

### Mice

Mice (BALB/c) of different age group i.e. infants, 2 months old and 4-5 week old females were procured from the animal house facility of National Institute of Virology, Pune, India. All animals were maintained according to the guidelines of Committee of Protection, Supervision and Control of Experiments on Animals. The project was approved by Indian GMO Research Information System (IGMORIS) and Institutional Biosafety Committee (IBSC).

### Cloning

**Figure 1:**
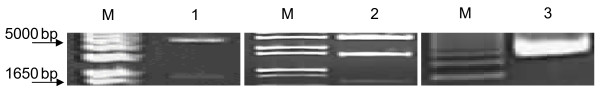
**Restriction analysis of the constructs**. Independent run gel documented in 1% Agarose in TAE buffer. M: 1 Kb+ Ladder (Invitrogen); 1: pCMV-E with SacI digested (Fragments: 4999 & 1812 bp); 2: pMS-E with VspI + NotI digested (Fragments: 2926 + 1551 + 703 bp); 3: pNIX-E (Undigested).

Plasmid used in the study was pAcGFP1-N1 (Clonetech, Takara). For the construction of (pCMV-E) construct, JEV propagated from tissue culture fluid of PS cells was used for RNA isolation (Trizol reagent, Invitrogen). The fragment of 2047 bp of partial C, prM and E gene [GenBank: EU372660] was amplified with Forward primer: 5'-GGTACCATGTGGCTCGAGAGCTTG-3' and Reverse primer: 5'-GGATCCTTTATTAAGCATGGACATTGGTCGCTA-3' employing Reverse Transcriptase PCR, using MMLV-RT and Platinum Taq. Start and stop codon (underlined) were included in forward and reverse primer respectively. Amplicon after cloning in pGEM^®^-T Easy cloning vector (Promega Corporation, Madison, USA) was excised using EcoRI restriction enzyme and cloned in EcoRI digested pAcGFP1-N1 expression vector. For (pMS-E), macrosialin promoter [GenBank: AF039399], was amplified with Forward primer: 5'-TATTAATGACCAAATCTACAGGGAGAACCC-3' and Reverse primer: 5'-AGCGCTAGATGCTCAGACCAGCTA-3' with VspI/Eco47III incorporated (underlined) and cloned in StrataClone™ PCR Cloning kit (Stratagene, USA). After digestion with VspI/Eco47III it was subcloned in similar digested pCMV-E construct and ligated. Devoid of promoter a negative control vector (pNIX-E) was constructed as described elsewhere [38]. Orientation and codon in-frame for all reconstructed clones were confirmed through restriction analysis (Figure 1) and sequencing.

Purified plasmids were prepared using EndoFree^® ^Plasmid Maxi Kit (Qiagen, Germany), according to manufacturer's instruction. The quality of plasmid was assessed using Nanodrop by light absorption at 260/280 nm ratio and by 1% agarose gel electrophoresis. All the plasmids were dissolved in sterile PBS for *in vivo *studies and nuclease free water for *ex vivo *studies.

### Western blot

Transfection was carried out with different constructs encoding E protein of JEV using Lipofectamine™2000 (Invitrogen, USA) with 2 μg of DNA as per manufacturer's instruction. Analysis for the blot was performed with 50 μg of cell lysate from RAW 264.7 cells. After 24 hours of transfection, cells were harvested, washed, mixed with an equal volume of 2× loading buffer and boiled for 10 min. Proteins were separated onto a discontinuous SDS-polyacrylamide gel with 5% stacking gel and 10% resolving gel and transferred to a nitrocellulose membrane (Amersham Biosciences, USA). The membrane was blocked by 5% skimmed milk powder in PBS and incubated with anti JEV monoclonal antibody [47] followed by goat anti-mouse IgG-HRP conjugate (Sigma). Bands were developed with diaminobenzidine tetrahydrochloride-H_2_O_2 _solution.

### Immunofluorescence assay

RAW264.7 cells grown on coverslips were transfected with pCMV-E, pMS-E and pNIX-E for 24 hours. The cells were fixed with 4% paraformaldehyde for 10 min and blocked with 1% BSA in PBS. The cells were permeabilized for detection of E protein, anti JEV monoclonal antibodies were used as primary antibody and probed with goat anti-mouse IgG FITC-conjugated Ab (Sigma, USA). The fluorescence was observed under confocal microscope.

### Mouse immunization and challenge experiments

Immunization studies were carried out in female inbred BALB/c mice aged between 4-5 weeks. Animals were divided into groups of 14, each for the following constructs: pCMV-E, pMS-E, pNIX-E, pCMV-E/MbrAg and PBS. All constructs were suspended in PBS with concentration of 1 μg/μl and antigen with 0.5 μg/μl with 100 μl inoculated. Mice were anesthesised and injected through i.m. route with 50 μl of constructs in both left and right quadriceps muscle. Animals in each group were given booster injection with the same concentration after 3 weeks of primary immunization and the next booster after 2 weeks. In pCMV-E/JE-MbrAg group, DNA construct followed by MbrAg as boosters were inoculated. After 7 weeks of immunization, all mice were challenged with lethal dose of JEV strain 733913 (100 LD_50_) through i.p. route followed by 1% starch by the i.c. route in order to breach the blood brain barrier [49]. These mice were observed for mortality for 6 weeks after challenge. Sera samples were collected by capillary through orbital sinus bleeding method at different time points and antibody response were assessed. To determine the 50% lethal dose (LD_50_) beforehand, groups of 11-12 weeks old mice were injected with i.p./i.c. starch route with 10 fold serial dilutions of virus.

### Enzyme Linked Immunosorbent Assay (ELISA)

Standard protocols for ELISA were used. Briefly, tissue culture derived JEV Ag (1 μg) was coated overnight in 50 μl of carbonate buffer. After blocking with 1% BSA in PBS, 100 μl of diluted mouse serum was added per well and incubated for 1 hr. After washing with PBST (PBS with 0.1% Tween 20), 100 μl of goat anti-mouse IgG-HRP conjugate (1:5000, Sigma) was added in each well. The colour was developed using the substrate orthophenyl-diamine (OPD) solution. The reaction was stopped with 100 μl of 4 N H_2_SO_4. _The absorbance was measured at 490 nm on a microplate reader (680 XR Microplate Reader). The end point titres of antibodies were determined as the reciprocal of highest dilution that gave an absorbance two times higher than that of nonimmune serum.

### Neutralization Assay

The virus neutralization assay was performed to assess the ability of produced antibody to neutralize the live JEV. As a positive serum JEV immune peritoneal fluid was used. The test was carried out with pooled mice sera of each group (pCMV-E, pMS-E, pNIX-E, pCMV-E/MbrAg and PBS) collected at day 0, 21, 36 and 51 before booster immunizations and complement inactivated. PS cells were seeded in 96 well plate with a density of 2 × 10^5 ^cells/ml in MEM supplemented with 10% FBS and incubated overnight. Fivefold serial dilution (1:10 to 1:1250) of serum along with controls was carried out in round bottomed microtitre plate (Nunc) and mixed with equal volume (60 μl) of 100 TCID_50 _of tissue culture pool of virus in MEM with 5% FCS. After incubation at 37°C for 1 hr, 100 μl of this antibody virus mixture was added to the preformed monolayer of cells and incubated for 3 days. Virus titration for determining the dose was challenge virus, incorporated in each plate. NT titre was expressed as reciprocal of serum dilution that resulted in more than 50% CPE [50].

### Cytokine profiling

Three weeks after the last immunization, splenocytes were harvested and resuspended at 2 × 10^5 ^cells/ml in RPMI 1640 (Gibco, USA) supplemented with 10% FBS (Gibco, USA) penicillin (100 IU/ml) and streptomycin (100 μg/ml). Each well of 24 well plate contained 4 × 10^5 ^cells in triplicate for each group. JE antigen (10 μg) was added to each well. As a positive control Con A (Sigma, USA) was used. After 72 hours of incubation at 37°C in 5% CO_2 _the supernatant was collected and centrifuged to remove the cell debris. That supernatant was stored in -70°C till tested.

The evaluation of Th1 and Th2 cytokine was performed by Cytometric Bead Array (CBA) (BD Biosciences) as per manufacturer's instruction. Briefly 50 μl of bead was mixed with the supernatant along with the given standards and incubated for 2 hours at RT in dark. The beads were washed and resuspended in 300 μl of wash buffer. The BD FACSAria™II instrument was then setup using BD FACSComp software and setup beads. Following acquisition the cytokine concentration was determined using the standard curve prepared.

### Lymphocyte proliferation assay

The assay was performed using tritiated thymidine incorporation as previously described [48]. Splenocytes were harvested from all the groups and resuspended at 2 × 10^5 ^cells/well in 96 well flat bottomed plate (Nunc). Cells were grown in RPMI 1640 (Gibco, USA) supplemented with 10% FBS with antibiotics and pulsed overnight with JEV antigen. Con A (Sigma, USA) was used as positive control. The assay was done in triplicates using three different concentrations. After 3 days the cells were pulsed with 1 μCi [^3^H] thymidine (BRIT) for 18 h. Cells were harvested onto GF/C (Whatman) filter disc and thymidine incorporation was measured with beta liquid scintillation counter (Tri-Carb^®^, PerkinElmer), as cpm. Data were represented as proliferation indices, calculated as: (Thymidine incorporated by cells in the presence of E protein/Thymidine incorporated in the absence of E protein)

### Statistical analysis

All of the test data were analyzed with PASW Statistics (version 18.0). Data were considered to be statistically significant when P < 0.05

## Results

### Western Blot Analysis

**Figure 2:**
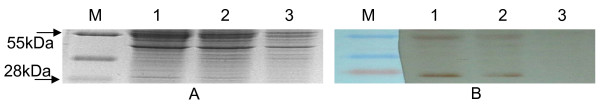
**Western Blot Analysis**. (A) 10% SDS-PAGE gel (B) Western blot analysis of the total cell lysates of the RAW 264.7 cells. M: PageRuler™ (Fermentas); 1: pCMV-E; 2: pMS-E; 3: pNIX-E. The blot shows expressed prM and E protein from different constructs after 24 hours of transfection.

**Figure 3:**
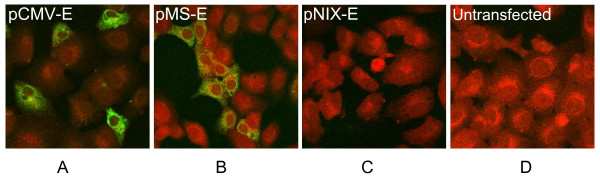
**Detection of E protein using IFA**. Expression of E protein in transfected RAW264.7 cells were detected 24 hrs post transfection. Figures (A) pCMV-E and (B) pMS-E confirms the expression of E protein whereas Fig. (C) pNIX-E and (D) Untransfected were used as a negative control.

After 24 hours of transfection similar amount of cell lysate were subjected to SDS-PAGE (Figure 2A) and Western blot analysis in all the wells. The monoclonal antibody against E protein reacted specifically with E protein of ~54 and prM of 20 kDa (Figure 2B, 1, 2). No visible band was observed in the lane of non transfected cells (Figure 2B, 3) indicative of absence of E and prM protein in the sample.

### Immunofluorescence assay

After the transfection, immunofluorescence analysis showed the green fluorescent cells which represented the expression of E gene of JEV (Figure 3A-B). No such signal was detected in the cells transfected with pNIX-E or untransfectd cells (Figure 3C-D).

### Mouse challenge experiments

Post immunization, no visible side effects were observed in the mice due to any of the construct used. To study the potency of vaccine, the immunized mice were challenged with lethal dose (100LD_50_) of JEV by previously reported method [48]. As JEV is not pathogenic by intra peritoneal (i.p.) route, following virus administration by i.p. route, blood brain barrier is breached by administration of 1% starch by intra cranial (i.c.) route. The mice were observed for mortality for 6 weeks post challenge.

Table 1 depicts that the mice immunized with pCMV-E shows 87.5% protection against lethal challenge of JEV. The newly constructed vaccine pMS-E was effective enough to give 75% protection. None of the mice survived after challenge from the negative control group pNIX-E and PBS. Significant level of difference was observed in groups of pCMV-E, pMS-E and pCMV-E/MbrAg in comparison to pNIX-E or PBS groups.

**Table 1:** Protection of mice challenged with lethal dose of Japanese encephalitis virus (strain 733913).

Plasmid Constructs	Number of mice challenged	Number of mice surviving	Protection (%)
pCMV-E	8	7	87.5
pMS-E	8	6	75
pCMV-E/MbrAg	8	7	87.5
pNIX-E	8	0	0
PBS	8	0	0

### ELISA

**Figure 4:**
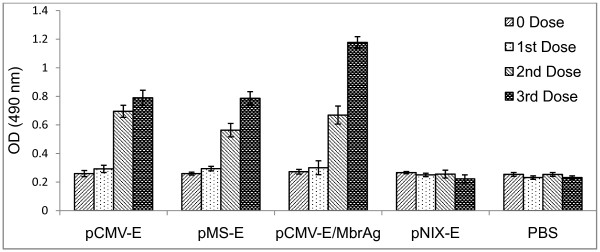
**ELISA**. Antibody response of BALB/c mice immunized with plasmid DNA of different constructs by intramuscular injection. First booster dose was given after 21 days of first immunization and second booster after 36 days. Serum samples were collected on the given days and stored at -70. Each column indicates the mean response ± SEM.

Antibody response was observed after immunization with different constructs through ELISA. Serum samples were collected from mice before every immunization and two weeks after the last immunization. Pooled sera were analyzed for the entire group in triplicate. The highest Ab titre was observed for the pCMV-E/MbrAg group, followed by pCMV-E and pMS-E, whereas only basal level of titre was obtained in pNIX-E and PBS inoculated mice. There were no significant differences (p > 0.05) in the Ab titre of all the groups in day zero and after the first dose, however significant increase in Ab titre after the second dose was observed in (pCMV-E, pMS-E and pCMV-E/MbrAg) groups which further increased after the next booster (Figure 4).

### Virus neutralization assay

**Figure 5:**
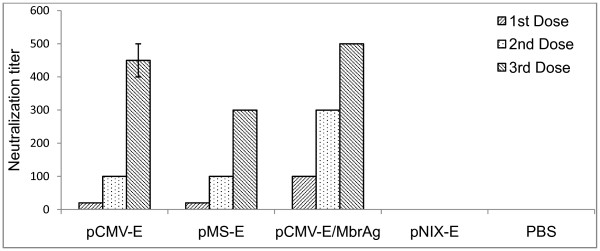
**Virus neutralization assay**. Neutralization assay of sera from mice immunized with different constructs. The highest dilution of mice sera that resulted into more than 50% CPE was considered. As a positive control JEV immune peritoneal fluid was used whereas peritoneal fluid from the non immunized mice was used as a negative control.

The virus neutralization test was performed to evaluate the ability of the constructs to elicit a neutralizing Ab response. Serum from the vaccinated groups showed considerable titres of JEV neutralizing Ab. Virus neutralizing Ab titres was observed to be 1:500 for the pCMV-E/MbrAg group, whereas for pCMV-E group it was 1:450. The newly constructed vaccine induced the Ab titre at 1:300 (Figure 5). This difference was consistent for the group and reproducible. No such neutralization Ab activity was observed for pNIX-E and PBS inoculated group.

### Cytokine profiling

**Figure 6:**
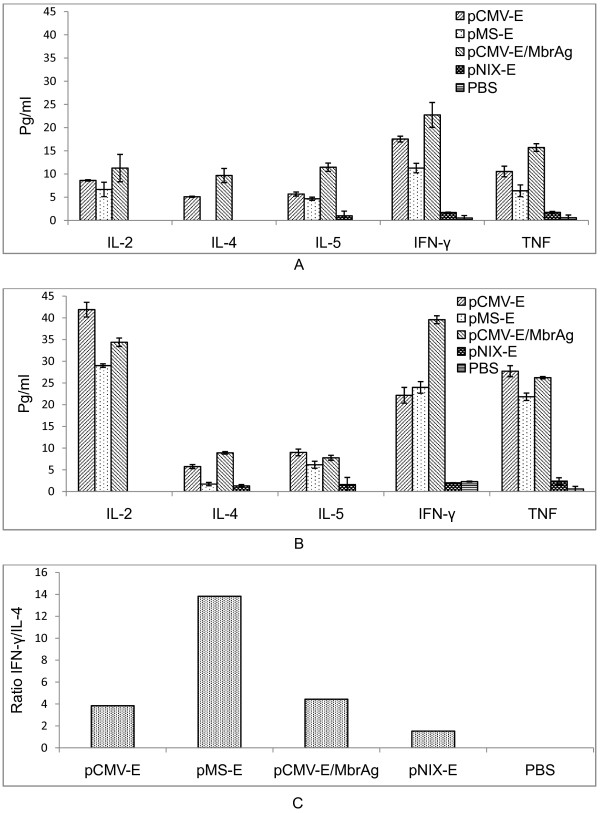
**Production of cytokines upon immunization of different constructs**. CBA was performed with the supernatant for Th1 and Th2 cytokines. The graph represents the concentration of cytokines TNF, IFN-γ, IL-2, IL-4 and IL-5. The data presented here are the means ± SEM of cytokine profile after (A) Second dose, (B) third dose and (C) the ratio of IFN-γ and IL-4 to show the skewness of immune response.

To characterize the immune response in different vaccinated groups, splenocytes were isolated after immunization protocol and the responses of T-cells were evaluated using mouse Th1/Th2 CBA system. This allowed simultaneous measurement of Th1 (IL-2, IFN-γ and TNF-α) and Th2 cytokines (IL-4 and IL-5) in antigen stimulated T-cells supernatant. After immunization in APC promoter group Th1 cytokines were notably high, while Th2 cytokines increased moderately. We observed significant increase in the cytokine levels compared to the pre-bleed level for any of the group. The level of cytokine was significantly higher in pCMV-E, pMS-E, pCMV-E/MbrAg immunized mice in comparison to the groups inoculated with pNIX-E and PBS. Hence the immune response was skewed towards Th1 type and only moderate towards Th2 (Figure 6).

### Lymphocyte proliferation assay

**Figure 7:**
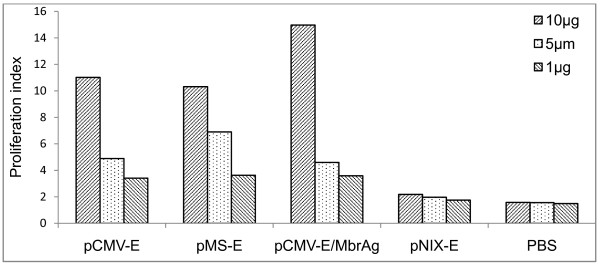
**Lymphocytes proliferation assay**. Lymphocytes from mice group immunized with different vaccine constructs were incubated with various amount of JEV Ag. After each dose the proliferative response was evaluated. Lymphocytes from unimmunized and pNIX-E were used as negative controls. Proliferation index were calculated from the means of experiments in triplicate.

The ability of splenocytes to proliferate when stimulated with the JEV Ag was analyzed through lymphocyte proliferation assay. We observed a significant increase in proliferation for pCMV-E, pMS-E, pCMV-E/MbrAg immunized mice in comparison to the pNIX-E and PBS inoculated groups (Figure 7). The response in proliferation increased on increasing the concentration of Ag upto 20 μg. Spleen from two mice per group was used for the assay.

## Discussion

Immune system has devised modalities to overcome nonspecific, autoreactive immune response while optimally maintaining the balance. Thus, immune response through antigen presentation by non-hematopoitic cells would actually be downregulated, while immune response generated through professional APC would be long term and balanced. Failure to have second signalling, which lack in non professional APC may lead to reduced immune response or even anergy [51]. The promoter of CMV is commonly used in mammalian expression system due to its strong activity in large varieties of cells [52]. The preference of gene expression is based on its application; in gene therapy constitutive expression is favoured [53]. On the contrary, to enhance the immune response of DNA vaccine, gene expression was restricted only to professional APC [54] and sometimes limited as a safety concern [55]. DC is considered to be the most potent and remained as highly preferred target cell. Although in a study, targeting DC alone was reported insufficient [35].

After *ex vivo *evaluation of promoters active in macrophages [38], we have selected the highest expressing constructs (macrosialin) and extended our study for *in vivo *analysis of macrosialin along with CMV promoter for comparison. JEV E protein expressed under these promoters was used for evaluation of concept in terms of antigen specific immune response. Induction of virus neutralizing antibodies, protection from lethal challenge and cytokine profile of immune splenocytes was studied and compared. E protein of JEV is considered to be a vital protein to target, because antibody against E protein has shown to neutralize JEV infectivity and also it is functionally important [12,56].

All the plasmid constructs were tested and confirmed to drive the expression of E gene of JEV before *in vivo *inoculation. In transfection experiment, Western blot and IFA analysis showed the constructs under study expressed E gene with high efficiency in RAW 264.7 cells. This indicated that the plasmid could be used further for subsequent *in vivo *experiments. Though we have shown the expression of E protein in RAW264.7 cells as a qualitative test, still the noticeably higher expression is seen with pCMV-E construct. We had shown similar results quantitatively in our earlier study using GFP reporter system [38].

To explore the applicability of these constructs as vaccine, groups of mice were vaccinated intramuscularly with recombinant plasmids. In addition, to evaluate prime boost models, administration of plasmid followed by MbrAg as a booster was used. All constructs, pCMV-E, pMS-E and pCMV-E/MbrAg (test groups) successfully led to the production of anti-E Ab. As measured by ELISA, the Ab produced in pCMV-E and pMS-E were nearly similar after the third dose, but increased significantly in pCMV-E/MbrAg groups. To measure the neutralizing abilities of these Ab, virus neutralization test was carried out. pCMV-E/MbrAg group showed the highest neutralizing Ab titre in all sera, whereas pCMV-E and pMS-E showed similar titre after the second dose, after the third dose the neutralizing Ab titre increased significantly in pCMV-E groups. The increase in Ab titre after subsequent dose shown by ELISA and neutralizing test was similar in comparison shown by other group (21).

For the evaluation of cellular response, lymphocyte proliferation assay was carried out. Significant proliferative response was observed in the splenocytes of vaccinated mice when compared with either pNIX-E or PBS inoculated mice. Similar proliferation indices were seen in the group of pCMV-E and pMS-E, whereas pCMV-E/MbrAg showed the highest response.

Cytokine has a major role to play in immune response against viruses, through direct antiviral activity as well as directing an array of immune responses to control the infection [57]. We studied the responses after immunization governed by different promoters through cytokine secretion profile of spleen cells. The result indicated that the magnitude of cytokine production was higher in pCMV-E in comparison to pMS-E at all times. The cytokine level for pCMV-E/MbrAg group increased dramatically after the second dose since it was boosted by MbrAg which acted as a better immunogen. This finding is consistent with previous studies which have demonstrated that DNA priming followed by protein boosting results in greater humoral response and mixed Th1/Th2 response with higher IFN-γ, IL-2 and IL-4 cytokines [58,59]. After the third dose increase in Th1 cytokine might have downregulated the Th2 cytokines. To evaluate the ratio of Th1 and Th2 responses, we observed the ratio of IFN-γ/IL-4, interestingly we observed macrosialin promoter highly skewed the response towards Th1 cytokine in comparison to CMV promoter. Our result for dominant Th1 response after i.m. inoculation of plasmid is in agreement with the earlier observation [21], moreover it also supports the previously observed Th1 biased response with APC targeted Ag delivery [54,60,61].

To assess the efficacy of vaccine in terms of protection, mice were challenged with lethal dose of JEV. We observed 87.5% protection in pCMV-E group and also for pCMV-E/MbrAg group, whereas it was 75% in pMS-E group. For protection to the host against JEV, neutralizing Ab plays a significant role [20]. For viral infections the importance of IFN-γ has been amply demonstrated [57]. Comparatively lower level of protection in pMS-E could be attributed to lower neutralizing Ab titre and lower IFN-γ levels (after second dose) and its non significant rise after the third dose. TNF is a proinflamatory cytokine considered efficient in stimulating DC maturation, migration and induction of proliferative and cytolytic activity of T cells and NK cells [62], for pMS-E lower TNF level was observed in comparison to pCMV-E or pCMV-E/MbrAg. With the acceptable difference in overall immune response in the constructs, it is considered as evidence of protection if the neutralizing titres are ≥ 1:10 [63].

In conclusion, CMV and Macrophage active promoter constructs resulted in successful expression of the E protein after intramuscular (i.m.) immunization. The expression level of pMS-E was lower than those obtained with the use of pCMV-E constructs but sufficient to induce protection in mouse model.

## Conclusions

In summary, we have demonstrated herein the *ex vivo *expression of E protein directed by CMV and Macrophage active promoter. When compared its activity in terms of immunity in mice model, response of pMS-E was lower than those obtained with the use of pCMV-E constructs but sufficient to induce protection in mouse model. With further study the use of macrosialin promoter could be an interesting alternative to the use of ubiquitous promoter, especially for the treatment of pathogen requiring dominant cellular immune response such as viral or anti-cancer vaccine.

## Competing interests

The authors declare that they have no competing interests.

## Authors' contributions

MFA has planned, designed and carried out all the experiments. MMG envisioned and supervised all the studies. Both the authors read and approved the final manuscript.
